# Inorganic phosphate: a link between processed food and exercise intolerance in the modern world

**DOI:** 10.1042/CS20250837

**Published:** 2026-08-14

**Authors:** John M. Giacona, Han-Kyul Kim, Wanpen Vongpatanasin

**Affiliations:** 1 Hypertension Section, Cardiology Division, Department of Internal Medicine, The University of Texas Southwestern Medical Center, 5323 Harry Hines Boulevard, Dallas, Texas, U.S.A.; 2 Department of Applied Clinical Research, The University of Texas Southwestern Medical Center, 5323 Harry Hines Boulevard, Dallas, Texas, U.S.A.; 3 Cardiology Division, Department of Internal Medicine, The University of Texas Southwestern Medical Center, 5323 Harry Hines Boulevard, Dallas, Texas, U.S.A.

**Keywords:** Cardiorespiratory Fitness, Exercise Intolerance, Inorganic Phosphate, Mitochondrial Function, Skeletal Muscle, Ultra-Processed Food

## Abstract

Physical inactivity and reduced cardiorespiratory fitness are major public health concerns worldwide. In recent decades, the modern western diet has undergone a dramatic shift toward ultra-processed foods (UPFs) that are rich in inorganic phosphate (Pi) additives used as preservatives, emulsifiers, leavening agents, and flavor enhancers. Unlike organic phosphorus found in natural foods, inorganic Pi additives are nearly completely absorbed in the gastrointestinal tract, contributing to habitual Pi intake that exceeds the recommended daily allowance by two- to three-fold in many western populations. Accumulating evidence suggests that this chronic Pi excess may impair skeletal muscle energetics and exercise capacity. In population-based cohort studies, higher serum Pi has been independently associated with reduced physical activity and increased sedentary time. Using 7-Tesla ^31^P magnetic resonance spectroscopy, higher dietary Pi intake has been correlated with lower resting muscle ATP synthesis and greater phosphocreatine depletion during exercise. Experimental studies have shown that a high-Pi diet reduces maximal oxygen uptake, treadmill endurance, and fat oxidation, and downregulates skeletal muscle fatty acid metabolism genes. At the cellular level, elevated Pi promotes muscle atrophy via autophagy and oxidative stress and reduces mitochondrial membrane potential. Endocrine mediators, including elevated fibroblast growth factor 23 and suppressed Klotho, may further contribute to muscle dysfunction. The present review synthesizes current evidence linking UPF consumption, dietary Pi excess, and exercise intolerance, with focus on skeletal muscle dysfunction, including impaired contractile mechanics, disrupted energy metabolism, and mitochondrial injury, as potential mechanisms, and highlights future directions for research and public health intervention.

## Introduction

Physical inactivity has become a global pandemic, with the age-standardized prevalence of inadequate physical activity reaching 31.3% in 2022 and rising in more than half of all countries surveyed [1]. Cardiorespiratory fitness (CRF), typically quantified as peak oxygen uptake (VO_2_max or VO_2_peak), is among the strongest predictors of cardiovascular and all-cause mortality. The 2016 American Heart Association (AHA) Scientific Statement designated CRF as a ‘clinical vital sign’ and a potentially stronger predictor of mortality than established risk factors including smoking, hypertension, and type 2 diabetes [2,3].

Despite widespread knowledge of the benefits of exercise, CRF has been declining in many populations over recent decades [4]. The reasons for this decline are multifactorial and include sedentary occupations, urbanization, and screen-based leisure activities. However, an underappreciated contributor may be the modern diet itself. Over the past several decades, the western diet has shifted dramatically toward so-called ultra-processed foods (UPFs). These industrial formulations, are made from cheap ingredients extracted or derived from whole foods and combined with additives, including flavor enhancers, emulsifiers, colorants, and preservatives [5]. In the United States, UPFs now account for approximately 57%–58% of total caloric intake in adults, a figure that has increased steadily from 53.5% in 2001–2002 [6,7]. Globally, UPF consumption ranges from 9% of total energy in Iran to 60% in the United States, with consumption exceeding 40% in Australia, Canada, and the United Kingdom [5]. Lack of universally accepted classification of UPFs has resulted in variability in the definition of UPF and its clinical implications. Currently, the NOVA classification is the predominant framework used in epidemiological studies linking food processing to health outcomes. However, the NOVA classification has been criticized for its reliance on qualitative descriptors and the presence of specific ingredients rather than nutrient content, which may introduce subjectivity and classification bias. Importantly, the present review focuses on inorganic phosphate (Pi) additives as a specific, objectively quantifiable component of processed foods that can be tested scientifically, rather than on the broader and more heterogeneous UPF category per se.

A growing body of evidence links UPF consumption to adverse health outcomes, including all-cause mortality, cardiovascular disease (CVD), and metabolic disease. However, these observational associations should be interpreted with caution, as UPF consumption is strongly patterned by socioeconomic status, and residual confounding may inflate effect estimates. Notably, negative control analyses have shown that UPF consumption is also associated with accidental death, an outcome for which no biological mechanism related to diet is plausible, suggesting that unmeasured confounders contribute to the observed associations [8]. Nevertheless, UPFs are a major source of Pi additives [9–11], a class of food additives that has received comparatively little attention in the context of exercise physiology and muscle health. In the present review, we synthesize the current evidence linking dietary Pi excess from UPF consumption to exercise intolerance and skeletal muscle dysfunction. We begin with the epidemiology of physical inactivity and its impact on CVD and mortality, discuss the health consequences of UPF consumption and the overconsumption of dietary Pi from inorganic sources, and present human, animal, and cellular data connecting Pi excess to impaired exercise capacity. We conclude with future directions for research and public health intervention.

## Physical inactivity and cardiorespiratory fitness: impact on cardiovascular disease and mortality

### The burden of physical inactivity

The scale of physical inactivity as a public health problem cannot be overstated. The 2024 Lancet Global Health pooled analysis of 507 population-based surveys across 163 countries (5.7 million participants) demonstrated that insufficient physical activity prevalence increased from 23.4% in 2000 to 31.3% in 2022, with the rate of increase accelerating in recent years [1]. Physical inactivity is now recognized as the fourth leading global risk factor for mortality, responsible for approximately 7.2% of all-cause deaths and 7.6% of CVD deaths worldwide [12]. The Global Burden of Disease 2019 study estimated 832,000 premature deaths and 15.7 million disability-adjusted life years attributable to low physical activity [13]. The economic burden is equally staggering: physical inactivity cost healthcare systems an estimated $53.8 billion international (INT) in direct costs and $13.7 billion in productivity losses in 2013 [14], and projections suggest that if current trends continue, 499.2 million new preventable non-communicable disease cases will occur globally by 2030, with direct healthcare costs exceeding INT $520 billion [15].

### Cardiorespiratory fitness as a predictor of mortality

The prognostic power of CRF has been established across diverse populations, regardless of age, race, or sex [3]. In a cohort of 122,007 patients from the Cleveland Clinic, low CRF is associated with a five-fold higher mortality risk compared with elite fitness (adjusted HR 5.04; 95% CI 4.10–6.20), exceeding the risk conferred by smoking (HR 1.41), diabetes (HR 1.40), or coronary artery disease (HR 1.29) [16]. Similarly, in a Veterans Affairs cohort of 750,302 individuals, the least fit had four-fold higher mortality compared with the most fit, with no upper limit of benefit observed even at extreme fitness levels [17]. The Cooper Center Longitudinal Study demonstrated that age-adjusted all-cause mortality rates were 3.4-fold higher in the least fit compared with the most fit men and 4.6-fold higher in the least fit women [18], with low fitness predicting increased mortality across short-, intermediate-, and long-term follow-up [19], and significantly improving CVD mortality risk reclassification when added to traditional risk factors [20].

Meta-analyses encompassing over 2.2 million participants consistently demonstrate that higher CRF, quantified as units of metabolic equivalents of task (MET), is associated with a 10%–17% reduction in all-cause mortality and an 18% reduction in heart failure incidence [21–23]. A comprehensive overview of 26 systematic reviews representing more than 20.9 million adults has confirmed that high versus low CRF is associated with an all-cause mortality HR of 0.47 (95% CI 0.39–0.56) and CVD mortality HR of 0.27 (95% CI 0.16–0.48) [23]. Using Fitness Registry and the Importance of Exercise National Database (FRIEND)-derived fitness categories, each 1-MET increment is associated with 12% lower all-cause, 16% lower CVD, and 14% lower cancer mortality [24]. Critically, the greatest mortality benefit occurs when moving from the least fit to the next-least-fit category, and improvements in CRF over time is associated with reduced mortality [3].

These data collectively establish CRF as among the most important modifiable predictors of longevity and cardiovascular health. Any factor that impairs CRF or contributes to exercise intolerance therefore has profound public health implications.

## Ultra-processed foods: a deleterious link to mortality and cardiovascular disease

### Definition and prevalence of ultra-processed foods

The NOVA classification system categorizes foods into four groups based on the extent and purpose of industrial processing: (1) unprocessed or minimally processed foods, (2) processed culinary ingredients, (3) processed foods, and (4) UPFs [5]. UPFs are distinguished from processed foods by their reliance on ingredients rarely or never used in home cooking, such as plant protein isolates, mechanically separated meat, and modified starches. Classes of sensory-related additives including colors, flavors, flavor enhancers, non-sugar sweeteners, and emulsifiers are also included. Common examples are carbonated soft drinks, reconstituted meat products, packaged snacks, industrial bread, instant soups, and ready-to-heat meals.

The dietary intake of UPFs has increased dramatically worldwide. In the United States, UPF consumption among adults increased from 53.5% to 57.0% of total energy intake between 2001 and 2018, while minimally processed food consumption declined from 32.7% to 27.4% [6]. UPFs comprise more than 50% of energy intake both at home and away from home [25]. Among US youths aged 2–19 years, UPF consumption increased from 61.4% to 67.0% of total energy over the same period. Globally, UPF sales have increased by 60%, especially in low-income countries, with increases of 40% in lower-middle-income countries, and nearly 20% in upper-middle-income countries between 2007 and 2022 [5].

### Ultra-processed foods, cardiovascular risk, and exercise intolerance

The association between UPF consumption and adverse health outcomes is robust and has been extensively reviewed elsewhere [5,26]. An updated dose-response meta-analysis shows a 15% increased risk of all-cause mortality associated with high-UPF consumption [27], and a comprehensive umbrella review of epidemiological meta-analyses reported 50% higher CVD-related mortality with the highest UPF intake [26]. The 2021 AHA Dietary Guidance Scientific Statement has recommended choosing minimally processed foods over UPFs [28], and the 2025 Lancet Series on UPFs concluded that the evidence linking UPF consumption to adverse health outcomes broadly fulfills seven of nine Bradford Hill criteria for causality [5].

Despite these strong associations with mortality and CVD, direct evidence linking UPF consumption to exercise capacity or VO_2_max remains limited. In the Framingham Offspring Study including 3003 adults, higher UPF consumption is inversely associated with physical activity level, with the proportion of participants reporting high physical activity declining from 58.5% in the lowest UPF quintile to 46.4% in the highest quintile [29]. In the NutriNet-Santé cohort of 44,551 adults, higher UPF consumption is similarly associated with lower physical activity levels [30].

While these cross-sectional associations do not prove causality, they raise the possibility that specific components within UPFs may directly impair exercise capacity. Among the many bioactive additives present in UPFs, Pi is of particular interest as a mechanistic link to exercise intolerance. This is because Pi additives are present in the majority of processed food products [9], are nearly completely absorbed in the gastrointestinal tract unlike organic phosphorus from whole foods [31], remain unregulated and undisclosed on food labels [9,32], and as detailed in the following sections, are supported by a growing body of mechanistic evidence directly linking Pi excess to impaired skeletal muscle energetics and exercise capacity.

## Sources and overconsumption of dietary phosphate

### Sources and bioavailability of dietary phosphate

Dietary Pi exists in three principal forms with markedly different oral bioavailability. Plant-derived organic phosphorus, dominated by non-absorbable phytates, has the lowest bioavailability (20%–50%), followed by animal-derived organic phosphorus bound in protein esters and phospholipids (40%–60%), which requires enzymatic hydrolysis for absorption [31]. Inorganic Pi additives, including phosphoric acid, sodium phosphate, sodium tripolyphosphate, and calcium phosphate, are already in dissociated ionic form and are absorbed nearly completely (80%–100%) without enzymatic processing. This difference matters clinically: in a randomized crossover trial, a vegetarian diet led to significantly lower phosphate absorption than a meat-based diet with similar total phosphorus content [33]. Moreover, a randomized double-blind crossover study showed that a high-inorganic-phosphorus meal produced higher serum and urinary phosphorus and significantly impaired endothelium-dependent vasodilation compared with a high-organic-phosphorus meal [34]. Food sources of organic and Pi can be found in Table 1.

**Table 1:** Sources of organic and inorganic phosphate

Phosphate source	Form	Examples	Estimated GI absorption	Key characteristics	References
Plant-based foods	Organic (phytate-bound)	Seeds, legumes, whole grains, nuts	20%–50%	Phosphorus stored as phytic acid; humans lack phytase enzyme for hydrolysis; lowest bioavailability	[31,105]
Animal-based foods	Organic (protein-bound)	Meat, poultry, fish, eggs, dairy	40%–60%	Phosphorus in intracellular organic compounds (phosphoproteins, phospholipids); requires enzymatic hydrolysis; dairy may have higher Pi:protein ratio than flesh proteins	[31,105]
Pi additives	Inorganic (dissociated salts/acids)	Sodium phosphate, calcium phosphate, phosphoric acid, sodium acid pyrophosphate, sodium tripolyphosphate, sodium hexametaphosphate	80%–100%	Already in ionic form; no enzymatic processing required; present in 44%–56% of US packaged foods; found in processed meats, frozen meals, baked goods, soft drinks, processed cheese	[9,11,32]

### Prevalence of phosphate additives and estimated dietary intake

Inorganic Pi additives are heavily used in food processing as preservatives, emulsifiers, stabilizers, leavening agents, and texture and flavor enhancers [5]. An analysis of 39,937 US food products found that 56% contained phosphate-based additives, with 21% containing more than one [9]. An earlier survey of 2394 best-selling branded grocery products found 44% contained phosphorus additives, with prevalence highest in prepared frozen foods (72%), dry food mixes (70%), and packaged meat (65%). Notably, additive-containing foods cost approximately $2.00 per day less than additive-free alternatives, creating a socioeconomic gradient in Pi exposure [10]. Similar patterns have been observed internationally: 36% of Finnish food products [11] and 44% of Australian grocery items contained Pi additives [35].

The recommended daily allowance (RDA) for phosphorus in the United States is 700 mg/day for adults [31]. However, the average American adult consumes approximately 1400 mg/day of phosphorus, nearly double the RDA [36], with food additives contributing an estimated additional 600–1000 mg/day of highly bioavailable inorganic Pi beyond natural food sources [10,37,38]. Controlled feeding studies have demonstrated that switching from a low-additive to an additive-enhanced diet increases daily phosphorus intake by approximately 606 mg (60%), accompanied by significant perturbations in bone and mineral metabolism [39]. National dietary surveys systematically underestimate true phosphorus intake because standard nutrient databases do not fully capture phosphorus from food additives. Compounding this problem, U.S. regulatory agencies do not require food manufacturers to disclose total phosphorus content on the Nutrition Facts label [9,32]. This regulatory gap means that consumers and clinicians alike are largely unaware of the true magnitude of Pi exposure from processed foods.

## Phosphate and exercise intolerance: direct phosphotoxicity

### Impaired exercise capacity and metabolic flexibility

The most compelling mechanistic evidence linking dietary Pi to exercise intolerance comes from controlled animal studies. Normal adult C57BL6 mice exhibit a significantly reduced maximal oxygen uptake (VO_2_max), treadmill duration, and spontaneous locomotor activity when fed with a high-Pi (HP; 2.0%) diet when compared with a normal-Pi (0.6%) diet for 12 weeks [40]. The impairment in exercise capacity induced by diet Pi loading is not explained by reduced cardiac function, as fractional shortening of left ventricle remains unchanged after a HP diet.

**Figure 1:**
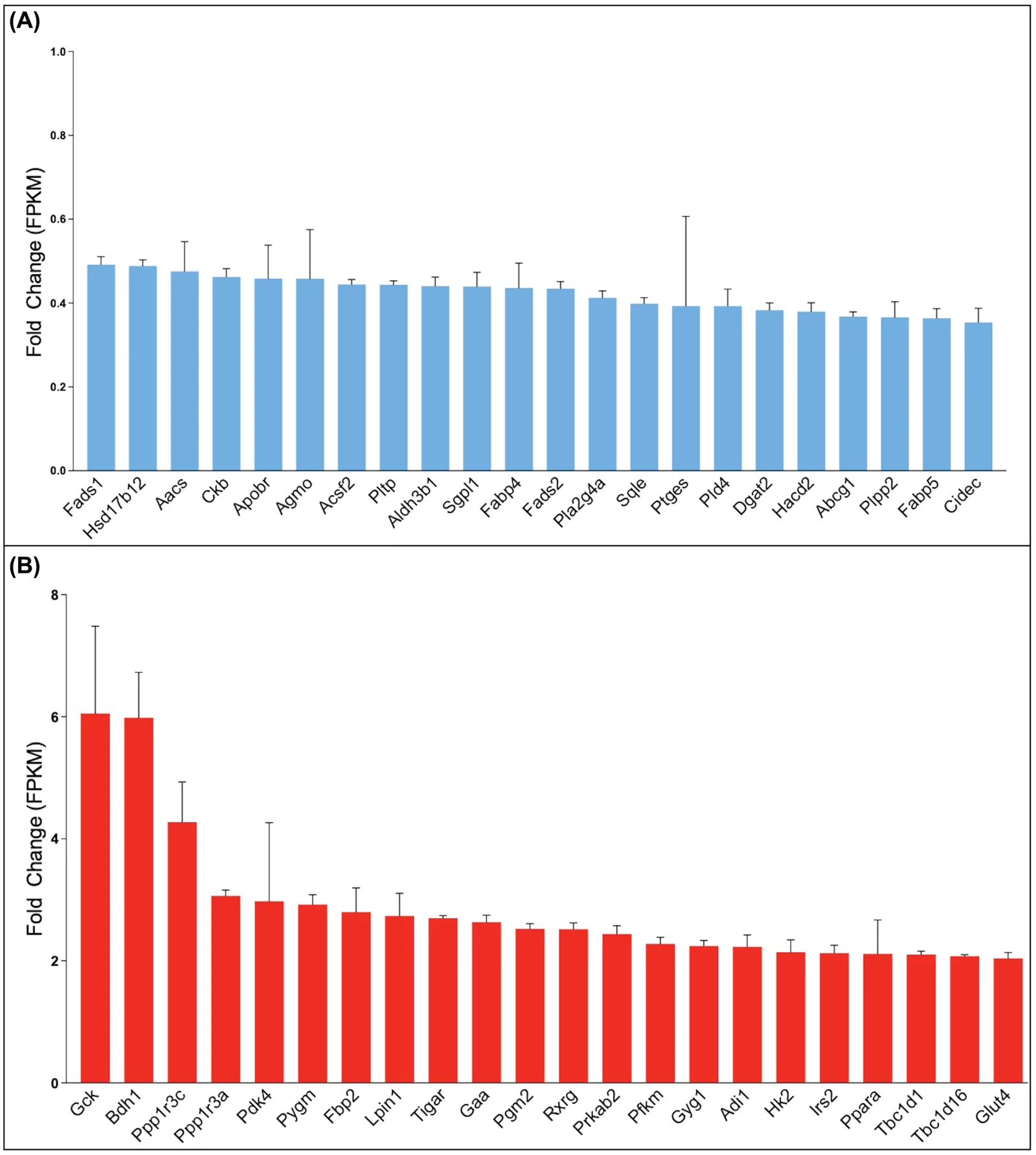
High-phosphate diet induces coordinated downregulation of lipid metabolism and upregulation of glucose metabolism genes in skeletal muscle Select high-throughput RNA seq data from gastrocnemius muscle of mice fed a high-phosphate (HP, 2.0% Pi) diet for 12 weeks. (**A**) Downregulated genes, the majority of which are involved in metabolism of lipids and lipoproteins. *Fads1*, encoding the Δ5 desaturase that catalyzes a rate-limiting step in long-chain polyunsaturated FA biosynthesis, showed the greatest reduction. Other notable downregulated genes include *Fabp4* and *Fabp5*. (**B**) Upregulated genes, many of which are involved in glycogen breakdown and glucose metabolism, including *Gck*, *Hk2*, *Irs2*, and *Glut4*. RNA seq data were generated as part of a previously published study [40].

Serum non-esterified fatty acid (FA) levels are lower in HP mice after exercise, suggesting impaired FA metabolism which is the critical pathway for energy homeostasis for low to moderate intensity exercise. The skeletal muscle gene expression profiling reveals downregulation of multiple genes involved in FA metabolism, FA synthesis, FA transport, and lipolysis. Among the downregulated genes, Fads1 is among the most strongly downregulated genes. FADS1 encodes a rate-limiting enzyme (Δ5 desaturase) needed to convert dietary FA into long-chain polyunsaturated FA like arachidonic acid and EPA, which are essential components of muscle membranes and inflammatory signaling [41]. Downregulation of Fads1 would be expected to impair the production of these bioactive lipids, which serve as key components of muscle membrane phospholipids and precursors to eicosanoid signaling molecules involved in inflammation and metabolic regulation [42,43]. Other downregulated genes involved in lipid metabolism include fatty-acid-binding protein 4 (*Fabp4*), *Fabp5*, hormone sensitive lipase (*Hsl*), fatty acid synthase (*Fasn*), peroxisome proliferator-activated receptor gamma (*Ppar-γ*), adiponectin (*Adipoq*), and patatin-like phospholipase domain-containing protein 3 (*Pnpla3*), as shown in Figure 1A. In contrast, many genes involved in glucose metabolism, including glucokinase (*Gck*), hexokinase 2 (*Hk2*), phosphoglycerate kinase 1 (*Pgk1*), insulin receptor substrate 2 (*Irs2*), lactate dehydrogenase subunit A (*Ldha*), and *Glut4*, are upregulated in the gastrocnemius muscle, as shown in Figure 1B. Critically, body weight, muscle mass, total body fat mass, and blood glucose are also unchanged, indicating that the exercise intolerance is attributable to a skeletal muscle-specific metabolic defect rather than obesity or factors outside skeletal muscle.

*In vitro* experiments using differentiated C2C12 myotubes exposed to HP media (1–3 mM) recapitulated the gene expression changes observed *in vivo*. Specifically, *Fabp4, Fabp5, Acsl5, Ucp2, Ppar-γ*, and *Hsl* were downregulated while *Eno3, Pgk1, Gpi,* and *Ldha* expression were upregulated after 2–10 days of incubation with media containing high phosphate milieu, which confirms a direct phosphotoxicity effect of Pi on the skeletal muscle cells. Since FA is the predominant energy substrate during prolonged low-to-moderate intensity exercise [44], the impairment in FA availability and oxidation in skeletal muscle during exercise may represent a critical mechanism underlying Pi-induced exercise intolerance by forcing earlier reliance on glycogen stores and premature fatigue onset during prolonged exercise.

Similar metabolic reprogramming has been demonstrated in the adipose tissue. Abuduli et al. demonstrated that hepatic lipogenic gene expression (including sterol regulatory element-binding protein-1c, *Fasn*, and acetyl-CoA carboxylase) are suppressed, while brown adipose tissue showed increased UCP1 and PGC-1α expression, suggesting enhanced thermogenesis [45]. Imi et al. confirmed that white adipose tissue exhibited decreased lipogenic and increased lipolytic gene expression during HP feeding [46]. Collectively, the metabolic changes in the skeletal muscle and adipose tissue may limit metabolic flexibility during exercise. Metabolic flexibility allows the body to choose fuel sources, i.e., switching between FA and carbohydrate metabolism, depending on substrate availability and exercise intensity and demand [47]. This ability to modify substrate oxidation in response to changes in nutrient availability is markedly attenuated in older and sedentary individuals [48–50] and, thus, diet Pi loading may contribute to accelerated aging phenotype via this mechanism.

### Mitochondrial dysfunction

In addition to dysregulated muscle metabolism, diet Pi intake may influence muscle mitochondrial function. Thus, disruption of normal intracellular Pi homeostasis, whether through deficiency or excess, could impair the ability of muscle mitochondria to match ATP production to exercise demand. When Pi is present in excess, the regulatory balance is disrupted. Excess Pi in the mitochondrial matrix disrupts pH gradients and increases the electrical potential across the membrane, causing electrons to ‘leak’ and generate harmful reactive oxygen species [51]. Pi is also a well-established activator of the mitochondrial permeability transition pore, promoting pore opening through depletion of matrix adenine nucleotides [52], enhanced oxidative stress [53], and at high concentrations, irreversible membrane permeabilization leading to collapse of transmembrane potential and arrest of ATP synthesis [54,55].

**Figure 2:**
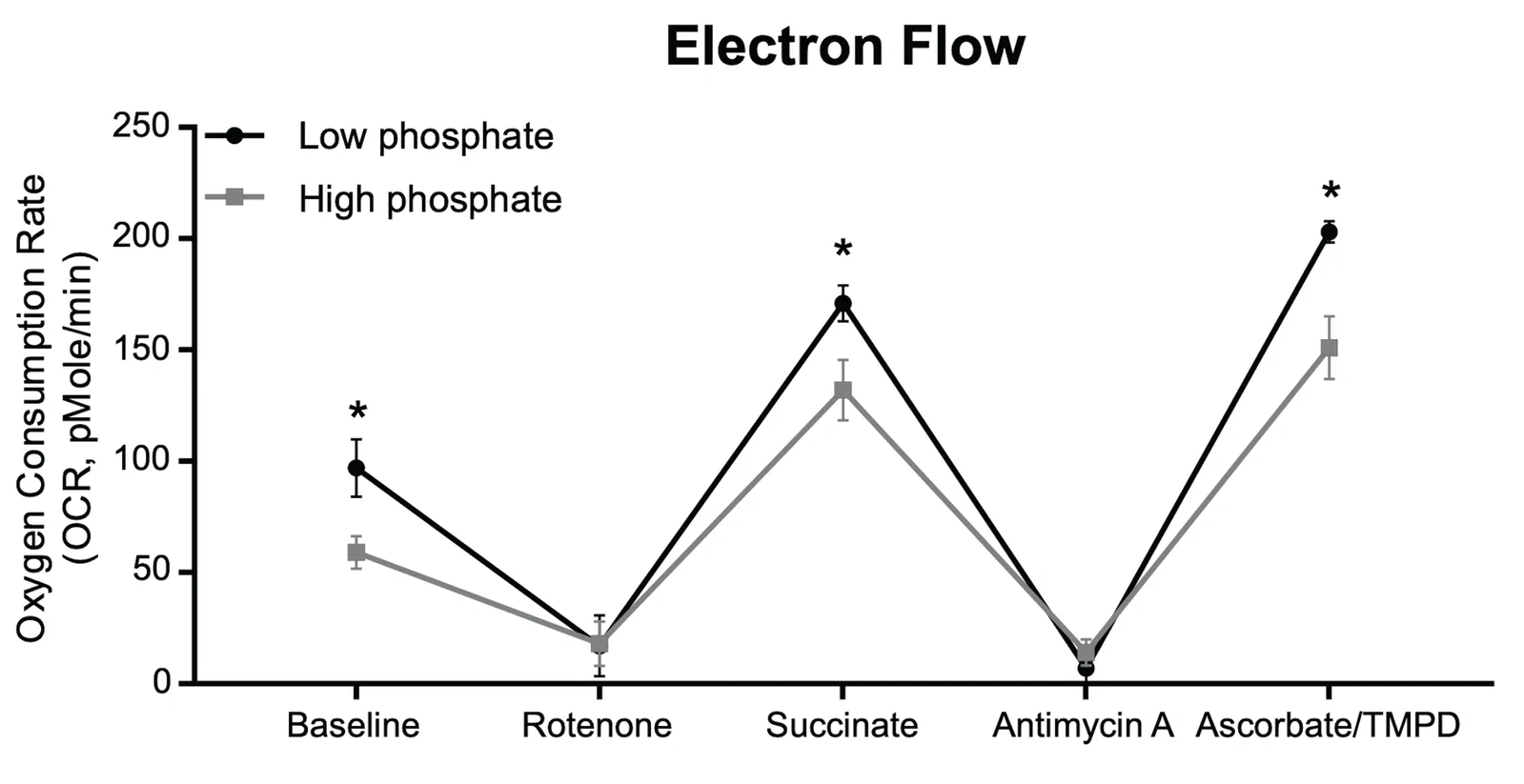
High-phosphate diet impairs electron transport chain-supported respiration across multiple entry points in skeletal muscle mitochondria OCR measured by electron flow assay in mitochondria isolated from gastrocnemius muscle of non-exercised mice fed a 0.6% Pi (low Pi) or 2.0% Pi (high Pi) diet for 12 weeks. Sequential injections of rotenone, succinate, antimycin A, and ascorbate/TMPD were used to assess respiration at individual electron entry points [40]. *P*<0.05 versus low Pi. Abbreviations: OCR, oxygen consumption rate; Pi, inorganic phosphate; TMPD, tetramethylphenylenediamine. OCR data were generated as part of a previously published study [40].

HP intake exerts multiple adverse effects on skeletal muscle mitochondrial function. *Ex vivo* studies in mitochondria isolated from gastrocnemius muscle demonstrated a significant impairment in mitochondrial function in mice on a HP diet as evidenced by reduced total oxygen consumption and respiratory control ratio, suggesting decreased mitochondrial capacity for substrate oxidation, ATP turnover, and a higher proton leak. To further localize the site of electron transport chain impairment, an electron flow assay was performed on mitochondria isolated from the same cohort of mice. Basal oxygen consumption rate (OCR) was measured first to assess overall mitochondrial respiration. Rotenone was then added to block Complex I, followed by succinate to drive electron entry at Complex II and respiration through Complexes III and IV. Antimycin A was then added to block Complex III, leaving only non-mitochondrial oxygen consumption. Finally, ascorbate/tetramethylphenylenediamine (TMPD) was added to deliver electrons directly to cytochrome c, isolating Complex IV-supported respiration. High-phosphate diet significantly reduced basal OCR, succinate-supported respiration, and ascorbate/TMPD-supported respiration, while oxygen consumption after rotenone and after antimycin A remained low and similar between groups (Figure 2). Because oxygen consumption remained low even when electrons were delivered directly to Complex IV (bypassing upstream complexes), the mitochondrial defect appears to extend to the final step of the electron transport chain. Furthermore, skeletal muscle mitochondria from the mice fed with HP diet also exhibited reduced oxygen consumption in response to palmitoylcarnitine, implicating impaired FA oxidation. The impairment is not explained by mitochondrial content, as mitochondrial DNA quantification is unaffected by dietary Pi intake.

### Muscle atrophy, senescence, and inflammation

Apart from impairment in mitochondrial function, Pi directly induces muscle atrophy [56]. *In vitro* studies in C2C12 cells exposed to HP environment showed a dose-dependent reduction in myotube size, fusion index, and expression of genes involved in myogenesis such as myogenin (MyoG). These changes were accompanied by increased production of reactive oxygen species (ROS), reduced mitochondrial membrane potential, and activation of Nrf2 signaling, a cellular stress response pathway through both ROS-dependent and p62-dependent mechanisms [56]. *In vivo*, mice fed a 2% Pi diet showed increased Nrf2 phosphorylation in gastrocnemius muscle, confirming the relevance of these pathways in intact animals. The deleterious role of HP milieu on the skeletal muscle may explain an accelerated sarcopenia with aging. Studies conducted in normal young and old C57BL6 mice up to 24 months old showed an age-related increase in serum Pi [57]. Furthermore, an inverse correlation between serum Pi and forelimb grip strength as well as skeletal muscle MyoG expression is observed while a positive correlation between serum Pi and muscle fibrosis as well as fibronectin gene expression is evident in these of mice [57]. Studies *in vitro* recapitulates the findings by demonstrating downregulation of MyoG and myocyte enhancer factor 2C (MEF2C) gene expression in the HP media up to 10 mM via inhibition of myogenic differentiation 1 (MyoD) transcriptional activity [57]. The inhibition of myogenic program is related to increased expression of histone deacetylase 1 (HDAC1), which binds directly to MyoD in undifferentiated myoblasts and removes acetyl groups that are required for MyoD’s transcriptional activity [58]. An independent study shows that even moderate phosphate elevations in normal physiological range (1.4–2.9 mM) significantly impairs expression of MyoG and MyoD via ERK phosphorylation [59]. More importantly, dietary Pi restriction from 0.6% to 0.2% prevents decline muscle strength and restores muscle MyoG and MEF2C expression *in vivo* in older mice, confirming a causal relationship [57].

In addition, increased extracellular Pi has been implicated in the development of myoblast senescence through integrin-linked kinase (ILK) overexpression, mTOR activation, and autophagy reduction [60]. ILK is a component of the transmembrane signaling complex that links the actin cytoskeleton to the extracellular matrix. Genetic deletion of ILK has been shown to extend lifespan in *Caenorhabditis elegans* and *Drosophila* [61]. Studies in older mice showed presence of hyperphosphatemia, which is accompanied by increased skeletal muscle expression of ILK. In addition, elevation in p62 protein expression, a marker of impaired autophagic flux, is observed in older mice. Similarly, *in vitro* studies have shown a deleterious role of high extracellular Pi in reducing autophagic flux as evidenced by reduction in the ratio LC3II/LC3I and increased p62 protein expression in C2C12 cells. The impairment in autophagy is prevented by silencing ILK expression.

Beyond autophagy and muscle aging, dietary Pi loading (3% Pi) upregulates expression of nuclear factor kappa-light-chain-enhancer of activated B cells (NFκB), interleukin 1 (IL1) and interleukin 6 (IL6), in hepatocytes in mice with normal kidney function. The upregulation of inflammatory pathway is accompanied by skeletal muscle wasting and increased expression of several genes involved in atrophy related programs such as muscle RING-finger protein 1 (Murf1) and Atrogin-1 [62]. Notably, a low-Pi diet ameliorates the phenotype of muscle wasting and reduced muscle strength.

### Disruption of muscle force generation

Intracellular Pi is regulated by a coordinated network of plasma membrane transporters (influx and efflux), intracellular organelle carriers, and anion channels that together maintain cytosolic Pi in the 1–5 mM range in skeletal muscle at rest. During exercise, intracellular Pi may rise to 30 mM. In normal physiological states, type III sodium-dependent phosphate cotransporters PiT1 (SLC20A1) and PiT2 (SLC20A2) are the principal channels regulating Pi influx in non-epithelial cells, including skeletal muscle. In addition, the ESCRT (endosomal sorting complexes required for transport) pathway is shown to regulate Pi sensing and PiT1 expression via internalization and degradation from the plasma membrane [63]. During muscle contraction intracellular Pi progressively increases due to ATP hydrolysis [64]. Evidence suggests that inorganic Pi directly impairs the myosin-actin cross-bridge cycle that underlies muscle force generation, resulting in muscle fatigue. *Ex vivo* studies in skeletal muscle fibers obtained via muscle biopsy shows that muscle from older adults (65–75 years) exhibits reduced force by 23%–37% and slower cross-bridge rate under fatigue conditions (30 mM Pi, pH 6.2) than muscle fibers from younger adults [65]. These studies establish that elevated intracellular Pi is a direct and potent inhibitor of muscle force generation, which is augmented in the presence of aging. Mechanisms underlying the findings are unknown. Elevated intracellular Pi is shown to reduce the Ca^2+^ sensitivity of the contractile apparatus, meaning higher Ca^2+^ concentrations are required to generate a given level of force [66]. In addition, intracellular Pi reduces Ca^2+^ release from sarcoplasmic reticulum during muscle nerve stimulation, resulting in reduced force generation [66]. Although, it is unclear whether dietary Pi loading affects intracellular levels of Pi in normal humans or rodents, it may a role in disease state that affects membrane integrity, such as chronic kidney disease (CKD).

### Deleterious role in CKD and Duchenne muscular dystrophy

Accelerated sarcopenia and muscle aging is the hallmark of patients with CKD. The phenotype of mice with dietary Pi excess resembles mouse models of CKD, including the adenine model and the Col4a3−/− (Alport) model [62]. The impairment in muscle grip strength and skeletal muscle wasting is independent of the bone-derived Pi-regulating hormone fibroblast growth factor 23 (FGF23)-FGF receptor 4 (FGFR4) signaling, which is shown to play an important role in cardiac myocyte remodeling during dietary Pi loading [62]. More importantly, a low-Pi diet ameliorated muscle wasting in the mice with CKD in a similar manner to mice with normal kidney function, indicating that direct phosphotoxicity as a primary driver of muscle aging and sarcopenia.

Hyperphosphatemia is also a well-recognized phenotype of dystrophin-deficient mdx mice, an animal model of Duchenne muscular dystrophy (DMD) [67,68]. The elevated serum Pi is postulated to be multifactorial including increased muscle membrane permeability related to absence of dystrophin which allows intracellular contents, such as Pi, to leak into the extracellular space. Skeletal muscle is the body’s largest reservoir of phosphate as approximately 85% of total body phosphorus resides in bone and muscle. Impaired kidney function may further exacerbate hyperphosphatemia via reduced renal clearance [67,69]. HP diet also exacerbates ectopic skeletal muscle calcification in the *mdx* mice [70], as evidenced by formation of hydroxyapatite (crystallized calcium phosphate) deposits within damaged fibers, which may further exacerbate muscle damage. On the other hand, dietary Pi restriction prevents ectopic calcification in *mdx* mice [68]. Studies in C2C12 cell culture shows direct effects of high-extracellular Pi environment (5 mM in the culture media) in driving osteogenic transdifferentiation of muscle cells (upregulation of Runx2 and osteocalcin genes). Although studies addressing serum Pi in patients with DMD are lacking, P31 NMR spectroscopy studies show evidence of higher levels of free intracellular Pi relative to ATP in the brain and skeletal muscle tissue, which may impair muscle force generation as described in the previous section [71–73].

### High-Pi diet augments the exercise pressor reflex

Beyond exercise capacity, high dietary Pi also affects cardiovascular responses during exercise. Dietary Pi loading triggers sympathetic overactivation and hypertensive responses in the model of simulated exercise in rodents [74]. Elevation of FGF23 is thought to be responsible for the finding by crossing the blood-brain barrier and activating FGFR4 in the brain [75]. Normally, muscle contraction activates muscle afferents which transmits signals to the brainstem and reflexively increases sympathetic nerve activity and blood pressure via a mechanism known as the exercise pressor reflex (EPR). The EPR is mediated by two important components: the muscle mechanoreflex, which is evoked by muscle deformation during contraction and the muscle metaboreflex, which is evoked by metabolites produced during muscle contraction. Dietary Pi loading induces overactivation of both the muscle mechano- and metaboreflex, which may further increase cardiac afterload during exercise and contribute to reduced peak VO2.

## Phosphate and exercise intolerance: human evidence

### Serum phosphate, physical activity, and muscle strength

The relationship between serum Pi and physical activity was examined in the Dallas Heart Study phase 2 (DHS-2), a multiethnic cohort study, in 1603 participants [40]. Higher serum Pi is independently associated with reduced time spent in moderate-to-vigorous physical activity and increased sedentary time after adjusting for age, sex, race, BMI, estimated glomerular filtration rate (eGFR), and other relevant covariates. Importantly, there is no association between serum Pi and left ventricular ejection fraction or volumes, indicating that the relationship is not mediated by cardiac dysfunction. These findings suggest that elevated Pi may impair exercise capacity through peripheral mechanisms, specifically effects on skeletal muscle, rather than through cardiac impairment.

Supporting these observations, population-based studies have linked serum Pi to reduced muscle strength. In a cross-sectional analysis of 7421 adults from the National Health and Nutrition Examination Survey (NHANES), higher levels of serum Pi are associated with lower quadriceps muscle strength. Elevated serum Pi levels even within normal physiological range is associated with a higher likelihood of dynapenia (low muscle strength without loss of muscle mass), particularly in adults older than 65 years [76]. Similarly, in the I-Lan Longitudinal Aging Study encompassing 2006 adults (mean age 65.5 years), higher serum Pi was significantly associated with weaker handgrip strength in both men and women, but not with reduced muscle mass [77]. More recently, Sun et al. reported in the Rugao Longitudinal Ageing Study that maximal tubular reabsorption of phosphate (TmP/GFR), a marker of renal Pi loading per nephron, is associated with higher risk of decreased grip strength (OR 2.03, 95% CI 1.20–3.49) and decreased Timed Up and Go performance (OR 1.49, 95% CI 1.05–2.12) over 4-year follow-up, with Mendelian randomization analysis supporting a causal relationship observed in the animal studies [78].

### Dietary phosphate and alterations in skeletal muscle energetics

Association between dietary Pi and muscle energy metabolism has also been examined in healthy humans [79]. Higher dietary phosphate intake, as captured by dietary survey (ASA24), is shown to be associated with lower resting ATP synthesis (in the reaction Pi + ADP => ATP) and greater phosphocreatine (PCr) depletion during plantar flexion exercise during 7-Tesla ^31^P magnetic resonance spectroscopy of the calf muscle, suggesting impaired muscle mitochondrial function. These associations remained significant after adjustment for factors known to affect skeletal muscle mitochondrial function and CRF, including age and eGFR. This is an important finding as normally PCr serves as energy buffer storage in the exercising muscles. Previous studies have shown a faster decline in PCr after exercise in older than in younger adults [80,81], which can be attenuated by exercise training [82,83]. Although the study is limited by small sample size and the cross-sectional design, these findings provide direct evidence that habitual dietary Pi excess is associated with impaired mitochondrial ATP production and abnormal muscle energetic responses during exercise.

**Figure 3:**
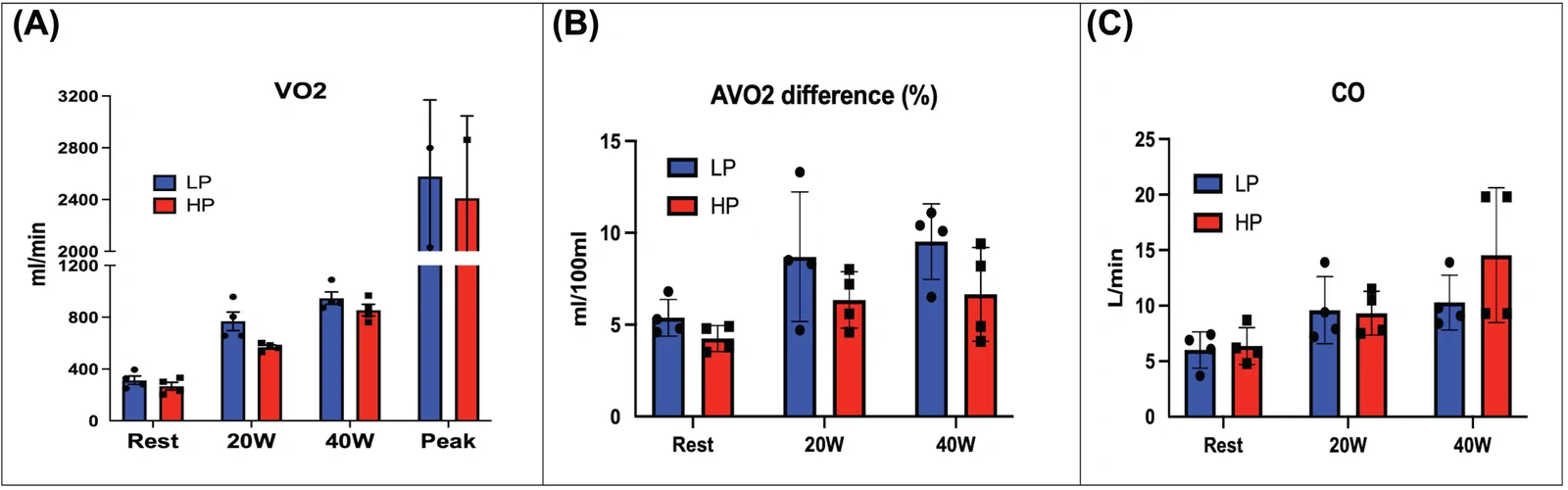
Reduced dietary phosphate intake increases exercise oxygen uptake and peripheral oxygen extraction in a pilot crossover study Summary data from a pilot randomized crossover study of four otherwise healthy adults comparing high phosphate intake with lower phosphate intake. Reducing total dietary phosphate intake from 1200 to 700 mg/day for 1-month increased (**A**) VO_2_ during cycle ergometer exercise (ANOVA *P* = 0.03 for diet effect). The increase in VO_2_ was paralleled by (**B**) higher arteriovenous oxygen difference, while (**C**) CO did not increase during exercise, consistent with greater peripheral oxygen extraction as the principal contributor to improved oxygen uptake. Twenty-four-hour urinary phosphate excretion decreased from 1082 ± 342 to 569 ± 132 mg/day, indicating effective reduction in phosphate loading (data not shown). Abbreviations: HP, high phosphate intake; LP, low phosphate intake; VO_2_, oxygen uptake; A-VO_2_ diff, arteriovenous oxygen difference; CO, cardiac output; Pi, inorganic phosphate.

To directly test whether reducing dietary Pi improves exercise capacity, we conducted a pilot randomized crossover study in four otherwise healthy subjects (age 25–60 years old) in which participants were randomized to receive sodium phosphate (NaPO_4_) capsules for 4 weeks during the HP phase to mimic usual US consumption (total Pi intake 1200 mg/d) versus sodium chloride capsules during the normal Pi phase (NP, total Pi intake 700 mg/d) for 4 weeks, while maintaining our study diet containing low Pi at RDA level (700 mg/d) for 8 weeks. VO_2_ during a cycle ergometer exercise was measured directly using metabolic cart (Medgraphics) after NP and HP phases in the same subjects. We found that lowering Pi intake for 1-month increased exercise VO_2_ at both submaximal level (at 20 and 40 watts) by 92–199 ml/min and at peak level by 167 ml/min (Figure 3A, ANOVA *P* = 0.03 for diet, and <0.01 for exercise). To put this in perspective, VO_2_ peak normally declines by about 12 ml/min per year in younger adults and 55 ml/min per year in older adults [84,85]. Therefore, the >10% improvement observed over just one month represents a change 3–10 times larger than the expected annual physiological decline. According to the Fick principle, oxygen uptake is determined by both cardiac output (CO) and peripheral oxygen extraction (arterio-venous O_2_ difference, A-VO_2_ diff), the latter is largely determined by mitochondrial oxidative capacity in the skeletal muscle during exercise [85,86]. In this regard, we also observed that the differences in exercise VO_2_ during HP and NP phases were driven by changes in A-VO_2_ diff and not by CO during exercise (Figure 3B,C). Thus, our pilot studies are consistent with animal studies showing *detrimental effects of Pi excess on exercise capacity at the skeletal muscle level*. Additional studies are being conducted in a larger clinical trial (NCT03234361) to verify these findings.

### Acute phosphate loading and exercise performance

It is important to note that a separate body of literature has examined short-term sodium phosphate supplementation as a potential supplemental aid in trained athletes. Several small studies have reported that 3–6 days of sodium phosphate loading (typically 3–5 g/day) can increase VO_2_max and delay fatigue [87,88], with proposed mechanisms including enhanced 2,3-diphosphoglycerate (2,3-DPG) production and rightward shift of the oxyhemoglobin dissociation curve. However, results have been inconsistent [89,90], with approximately half of controlled studies showing no performance benefit. A more recent study of 16 well-trained cyclists found no improvement in 30-km time-trial performance after 4 days of sodium phosphate loading [91]. These acute loading studies differ fundamentally from chronic dietary Pi excess: the former involves short-term supraphysiologic supplementation in already fit athletes, whereas the latter concerns long-term moderate overload from processed food additives in the general population. The observation of occasional short-term benefit does not contradict the evidence that chronic HP diets impair muscle function, and may instead reflect the importance of Pi dose, duration, and hormonal adaptation in determining the net effect on exercise capacity.

### Endocrine mediators: FGF23 and Klotho

As mentioned earlier, dietary Pi loading triggers release of the phosphaturic hormone FGF23 from osteocytes and osteoblasts to minimize Pi overload [92]. These endocrine changes may contribute to the link between dietary Pi and skeletal muscle dysfunction.

Skeletal muscle expresses FGF receptors (FGFR1-4) establishing the molecular machinery for FGF23 signaling [93]. Whether FGF23 directly impairs skeletal muscle function remains debated. While FGF23 is not shown to alter C2C12 myoblast proliferation, differentiation, or *ex vivo* contractility in some studies, others demonstrated that FGF23 reduced expression of myogenic regulatory factors and myosin heavy chain in human skeletal muscle satellite cells [94] and induced premature senescence in the skeletal muscle-derived mesenchymal stem cells [95]. These discrepancies may reflect differences in cell type, Klotho availability, or duration of exposure. Notably, Heitman et al. more recently identified skeletal muscle as a novel source of FGF23 production in response to phosphate elevations, with skeletal muscle-specific Fgf23 deletion resulting in elevated serum phosphate during HP feeding, beyond the bone production of FGF23 [96]. Importantly, whether muscle-derived FGF23 exerts paracrine effects on muscle function remains to be determined.

The relationship between FGF23 and muscle function appears context dependent. Exercise-stimulated FGF23 enhanced exercise performance and mitochondrial function in mice by reducing excess ROS and increasing PPAR-δ and citrate synthase activity [97]. In contrast, chronic pathologic FGF23 elevation is associated with adverse muscle outcomes: higher FGF23 was associated with lower knee extension strength and aerobic capacity in adults with normal kidney function [98], 38% higher odds of frailty per doubling of FGF23 in the Cardiovascular Health Study [99], and prevalent frailty and incident falls in SPRINT participants with CKD [100].

In addition of FGF23, higher dietary Pi is shown to downregulate α-Klotho, an anti-aging protein, which plays an important role in maintaining Pi homeostasis in conjunction with FGF23 [93]. Studies in humans have shown an association between dietary Pi loading and impaired muscle energetics, as evidenced by PCr depletion and ADP accumulation post-exercise in healthy humans [79]. Higher circulating Klotho level is linked to higher grip strength in the InCHIANTI study [101]. It also predicts higher baseline knee extension strength and less decline over 4 years in the health aging, body, and composition study [102], and Mendelian randomization analysis found that genetically predicted higher α-Klotho was causally associated with lower risk of low grip strength and lower frailty index [103]. Exercise itself may partially counteract Klotho suppression, as chronic exercise training has been shown to increase Klotho levels.

In addition to FGF23 and α-Klotho, fibroblast growth factor 21 (FGF21) may represent another endocrine mediator linking phosphate excess to systemic complications. FGF21 is a liver-derived hormone that binds to the βKlotho–FGFR complex in the central nervous system to activate the sympathetic nervous system and the hypothalamus–pituitary–adrenal axis. Like FGF23, circulating FGF21 levels rise progressively with declining renal function. Nakano et al. demonstrated in a CKD mouse model induced by unilateral nephrectomy and a high-phosphate diet that sympathetic activation and glucocorticoid elevation were largely dependent on FGF21, as Fgf21-knockout CKD mice escaped these complications despite dying earlier [104]. While this study was performed in CKD rather than in animals with normal kidney function, it raises the possibility that some systemic consequences of phosphate overload, particularly sympathetic overactivation, may be mediated through FGF21 in addition to FGF23. Whether FGF21 contributes to phosphate-induced skeletal muscle dysfunction or exercise intolerance warrants further investigation.

## Conclusions and future directions

**Figure 4:**
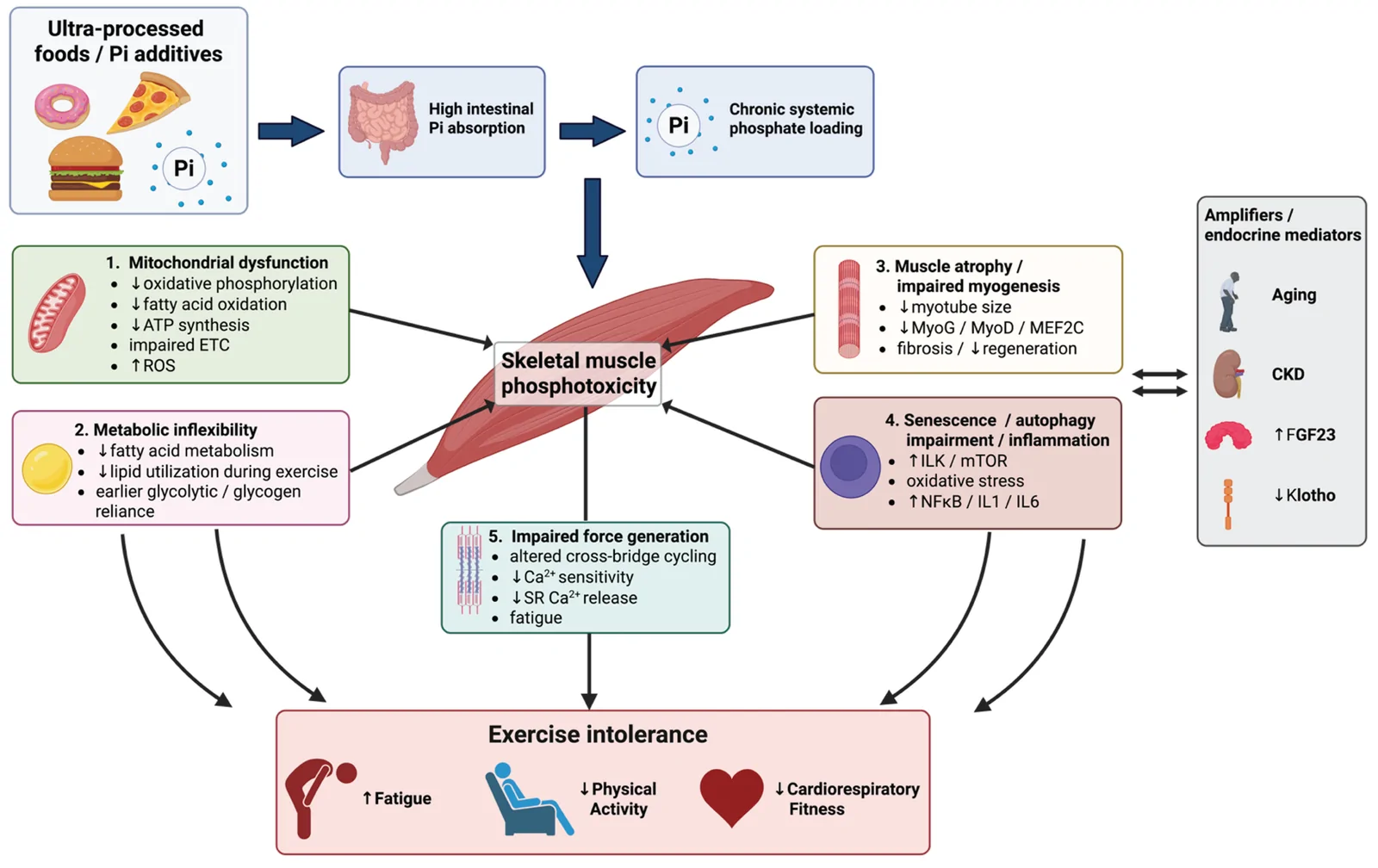
Proposed mechanisms linking excess dietary inorganic phosphate to skeletal muscle dysfunction and exercise intolerance UPFs are a major source of highly bioavailable Pi additives, which can increase systemic phosphate loading. Chronic Pi excess may impair skeletal muscle function through multiple, potentially overlapping mechanisms, including mitochondrial dysfunction with reduced oxidative phosphorylation and ATP synthesis, impaired FA oxidation and metabolic flexibility, activation of oxidative stress pathways, muscle atrophy, and impaired myogenic differentiation, cellular senescence and autophagy impairment, inflammation, and reduced muscle force generation. These effects may be further modified or amplified by aging, CKD, and endocrine responses to phosphate excess, including increased FGF23 and reduced Klotho. Collectively, these mechanisms may contribute to reduced exercise capacity, earlier fatigue, lower physical activity, and declining CRF. Abbreviations: ATP, adenosine triphosphate; CKD, chronic kidney disease; FGF23, fibroblast growth factor 23; Pi, inorganic phosphate.

Physical inactivity and reduced CRF remain major global health challenges, with dietary inorganic Pi emerging as a plausible and underappreciated contributor to exercise intolerance. HP intake from processed food additives, which are nearly completely absorbed and largely unregulated on food labels, results in chronic Pi excess that far exceeds recommended levels in many western populations. Accumulating evidence indicates that this Pi excess impairs skeletal muscle function through multiple mechanisms, including increased mitochondrial oxidative stress, impaired ATP synthesis, suppression of muscle FA synthesis and oxidation, and activation of atrophy and senescence pathways. Endocrine mediators of Pi toxicity, including elevated FGF23 and suppressed Klotho, may further contribute to skeletal muscle dysfunction. These proposed mechanisms are shown in Figure 4.

From a public health perspective, these data support the need for disclosure of total phosphorus content on food labels, which would enable consumers and clinicians to make informed dietary choices and facilitate future research. While confirmatory randomized controlled trials are needed to establish causality between dietary Pi reduction and improved exercise capacity in humans, the low risk of improved labeling provides a reasonable basis for this measure independent of ongoing trials. Dietary strategies aimed at reducing inorganic Pi additive intake, such as choosing minimally processed foods and avoiding phosphoric acid-containing beverages, may represent a simple and modifiable approach to preserving skeletal muscle function and CRF. The benefits of limiting Pi additives may extend beyond the general population to particularly vulnerable groups, including patients with CKD or DMD, in whom phosphate toxicity is amplified. Overall, the present review highlights dietary inorganic Pi as a novel and potentially modifiable link between the modern processed food environment and the global epidemic of physical inactivity and exercise intolerance.

## Data Availability

Data sharing is not applicable to this article.

## Open Access

Open access for this article was enabled by the participation of University of Texas Southwestern Medical Center in an all-inclusive Read & Publish agreement with Portland Press and the Biochemical Society.

## Abbreviations

ATP: Adenosine triphosphate
Ca^2+^: Calcium ion
CKD: Chronic kidney disease
CO: Cardiac output
CRF: Cardiorespiratory fitness
CVD: Cardiovascular disease
EPR: Exercise pressor reflex
eGFR: Estimated glomerular filtration rate
Fabp4: Fatty-acid-binding protein 4
Fasn: Fatty acid synthase
FGF23: Fibroblast growth factor 23
FGFR4: Fibroblast growth factor receptor 4
Gck: Glucokinase
Hk2: Hexokinase 2
HP: High-Pi
Hsl: Hormone sensitive lipase
ILK: Integrin-linked kinase
Irs2: Insulin receptor substrate 2
Ldha: Lactate dehydrogenase subunit A
MEF2C: Myocyte enhancer factor 2C
MET: Metabolic equivalents of task
MyoD: Myogenic differentiation 1
MyoG: Myogenin
OCR: Oxygen consumption rate
PCr: Phosphocreatine
Pgk1: Phosphoglycerate kinase 1
Pi: Inorganic phosphate
Ppar: Peroxisome proliferator-activated receptor gamma
RDA: Recommended daily allowance
ROS: Reactive oxygen species
TMPD: Tetramethylphenylenediamine
UPF: Ultra-processed food
